# Case Report: mNGS-negative *Strongyloides* hyperinfection syndrome with small bowel obstruction as the initial manifestation in a glucocorticoid-exposed COPD patient: a fatal case

**DOI:** 10.3389/fimmu.2026.1940986

**Published:** 2026-10-06

**Authors:** Jiaqi Su, Yingying Lu, Yuanzhi Xia, Chunhong Xie, Jinyan Kai, Lianjiang Huang

**Affiliations:** Department of Clinical Laboratory, The Second Affiliated Hospital of Xiamen Medical College, Xiamen, Fujian, China

**Keywords:** chronic obstructive pulmonary disease, Hyperinfection syndrome, Intestinal Obstruction, metagenomic next-generation sequencing, *Strongyloides stercoralis*

## Abstract

**Background:**

*Strongyloides* hyperinfection syndrome (SHS) is a life-threatening condition in immunocompromised hosts with an extremely high mortality rate. Metagenomic next-generation sequencing (mNGS) demonstrates limited sensitivity for parasitic detection, and its clinical value remains to be fully elucidated. The conventional microscopy remains an indispensable tool for the diagnosis of parasitic infections.

**Case description:**

A 73-year-old male patient with a history of chronic obstructive pulmonary disease (COPD), diabetes mellitus, hypertension, and cerebral infarction presented with abdominal pain and distension for 7 days and no bowel movement for 3 days. He was diagnosed with incomplete small bowel obstruction at an outside hospital and treated with fasting, enemas, meropenem, and methylprednisolone. He was subsequently transferred to our hospital due to acute lower respiratory tract infection. Chest radiography revealed bilateral diffuse infiltrates, and his condition rapidly deteriorated, requiring admission to the intensive care unit (ICU), endotracheal intubation, mechanical ventilation, and continuous renal replacement therapy (CRRT). Stool smear and retrospective sputum examination both identified larvae of Strongyloides stercoralis. However, mNGS of sputum and bronchoalveolar lavage fluid (BALF) obtained upon admission failed to detect any parasitic sequences. The patient was treated sequentially with albendazole and ivermectin, in combination with broad-spectrum antibacterial, antifungal, and antiviral therapy. He ultimately died on February 11, 2025, due to severe pneumonia, sepsis, and bone marrow suppression.

**Conclusion:**

For immunocompromised hosts with multiple risk factors, particularly those exposed to glucocorticoids, active parasitic microscopic examination of stool and respiratory secretions should be pursued when unexplained pulmonary infiltrates coexist with gastrointestinal symptoms, even in the setting of negative mNGS results, to avoid missed diagnosis.

## Introduction

*Strongyloides stercoralis* (*S. stercoralis*) is a soil-transmitted nematode predominantly distributed in tropical and subtropical regions, with endemic presence also reported in humid areas of southern China (1). This parasite possesses the unique capacity of autoinfection, wherein rhabditiform larvae can directly develop into infective filariform larvae within the host, bypassing the external soil phase and thereby enabling persistent long-term infection. In immunocompetent hosts, chronic infection typically manifests as an asymptomatic intestinal carrier state (2). When host immune function is compromised, the parasite can proliferate massively through an accelerated autoinfection cycle, leading to *Strongyloides* hyperinfection syndrome (SHS) or even disseminated infection involving the lungs, brain, and other organs (3–5). SHS is a life-threatening condition with rapid progression, and mortality can reach up to 87% when there is dissemination (6). Underlying diseases such as diabetes mellitus can induce systemic immune dysfunction (7), thereby increasing susceptibility to SHS.

This case report describes a patient with SHS presenting with small bowel obstruction as the initial manifestation. The aim is to emphasize that for immunocompromised hosts with risk factors, when unexplained pulmonary infiltrates coexist with gastrointestinal symptoms, active parasitic microscopic examination should be pursued even in the setting of negative mNGS results, to avoid missed diagnosis and delayed treatment.

## Case presentation

A 73-year-old male patient with a past medical history of COPD, diabetes mellitus, hypertension, and cerebral infarction. He presented with abdominal pain and distension for 7 days and no bowel movement for 3 days. He was evaluated at an outside hospital on January 21, 2025, where he was diagnosed with incomplete small bowel obstruction. Treatment included fasting, enemas, meropenem for infection control, and methylprednisolone for anti-inflammatory therapy (specific dosage and duration unknown). On January 26, 2025, the patient was transferred to the respiratory department of our hospital for acute lower respiratory tract infection secondary to COPD.

On admission, the patient was alert but appeared acutely ill and malnourished. His vital signs were temperature 36.4 °C, pulse 128 beats/min, respiration 21 breaths/min, and blood pressure 135/67 mmHg, with oxygen saturation of 79% on room air. He was brought in on a stretcher and was unable to walk or transfer independently. He had dyspnea, palpitations, and tachypnea at rest, with cough and yellow-white sputum occasionally streaked with blood. He required a high sitting position to breathe at night. He had not had a bowel movement for nearly 2 days, with poor appetite and sleep. Physical examination revealed pale skin, lips, and mucous membranes, with no enlarged superficial lymph nodes and no pharyngeal congestion or tonsillar enlargement. A 6 × 5 cm stage 1 pressure injury was present over the sacrum. His body weight had not been monitored before transfer, so BMI could not be calculated. He was dependent on caregivers for activities of daily living. Chest radiograph in the anteroposterior view revealed bilateral diffuse infiltrates (**Figure 1**). Due to severe pulmonary symptoms, the patient was transferred to the intensive care unit (ICU). On February 1, 2025, endotracheal intubation was performed and mechanical ventilation was initiated. On February 3, 2025, continuous renal replacement therapy (CRRT) was started. On January 28, 2025, bronchoscopy revealed bloody viscous sputum in both upper lung segments. Laboratory findings are presented in **Table 1**. On January 29, 2025, the patient had his first bowel movement. The stool smear examination identified *S. stercoralis*. Retrospective re-examination of the sputum specimen collected on January 27 also detected *S. stercoralis* larvae (**Figure 2**), and a diagnosis of SHS was established. mNGS of the sputum collected on January 27 and bronchoalveolar lavage fluid (BALF) collected on February 3 did not detect any parasitic sequences (**Supplementary Table 1**). Sputum culture from January 27 yielded growth of *Candida* and filamentous fungi. Multiple blood cultures performed during hospitalization were all negative.

**Table 1:** Laboratory test results during the patient's hospital stay.

Date	WBC×10^9^/L	RBC×10^12^/L	HGB g/L	PLT×10^9^/L	Ret%	Ret×10^9^/L	CRP mg/L	PCT ng/mL	ALB g/L	ALT IU/L	AST IU/L	BNP pg/mL	Ferritin ng/mL	Serum G test pg/mL	Serum GM (ELISA)	BALF GM (ELISA)	HIV (CLIA)
26.1.2025	11.67	2.46	76	253			18.20		32.80	68.0	52.7	86.2					
27.1.2025	12.28	1.95	61	227	2.45	48.5	9.43	0.55						275.786	-ve		-ve
29.1.2025	9.62	1.84	55	131			7.33					475.1					
30.1.2025	11.46	2.29	71	110				0.33				220.3					
31.1.2025	11.99	2.42	72	114			38.35	1.09	32.10	36.0	41.9	245.8					
1.2.2025	13.54	1.98	61	80			22.14	17.21				353.8	177.3	<37.50	-ve	+ve	
2.2.2025	15.65	1.99	62	71	2.80	55.7	15.55	20.23	26.20	35.9	50.0	150.1					
3.2.2025	13.38	1.72	53	49	1.92	33.0	8.92	19.10				91.3					
4.2.2025	8.34	1.97	59	41	1.50	29.5	9.51	5.82				70.6					
5.2.2025	2.89	1.74	51	29	1.19	20.7	51.93	3.90	40.90	70.1	148.4				-ve	+ve	
6.2.2025	0.73	1.65	49	13	1.20	19.8	93.41	2.74		877.8	2015.5	584.9					
7.2.2025	0.21	1.74	52	20	0.96	16.7	88.60	2.73	35.30	2024.1	7477.0	968.0	>1500				
8.2.2025	0.12	1.04	32	7	0.75	7.8	93.49	1.96	32.20	1650.1	2714.6	444.1					
9.2.2025	0.06	0.96	31	66	0.42	4.0	27.00	1.47	35.90	367.5	498.7	480.3					
10.2.2025	0.10	1.40	43	13	1.03	14.4	33.17	1.17	33.40	284.4	181.6	424.3					

WBC, white blood cell; RBC, red blood cell; HGB, hemoglobin; PLT, platelet; Ret, reticulocyte; CRP, C-reactive protein; PCT, procalcitonin; ALB, albumin; ALT, alanine aminotransferase; AST, aspartate aminotransferase; BNP, B-type natriuretic peptide; G test, (1,3)-β-D-glucan test; GM, Galactomannan; BALF, Bronchoalveolar Lavage Fluid; ELISA, Enzyme-Linked Immunosorbent Assay; HIV, Human Immunodeficiency Virus; CLIA, Chemiluminescence Immunoassay; -ve, negative; +ve, positive.

**Figure 1:**
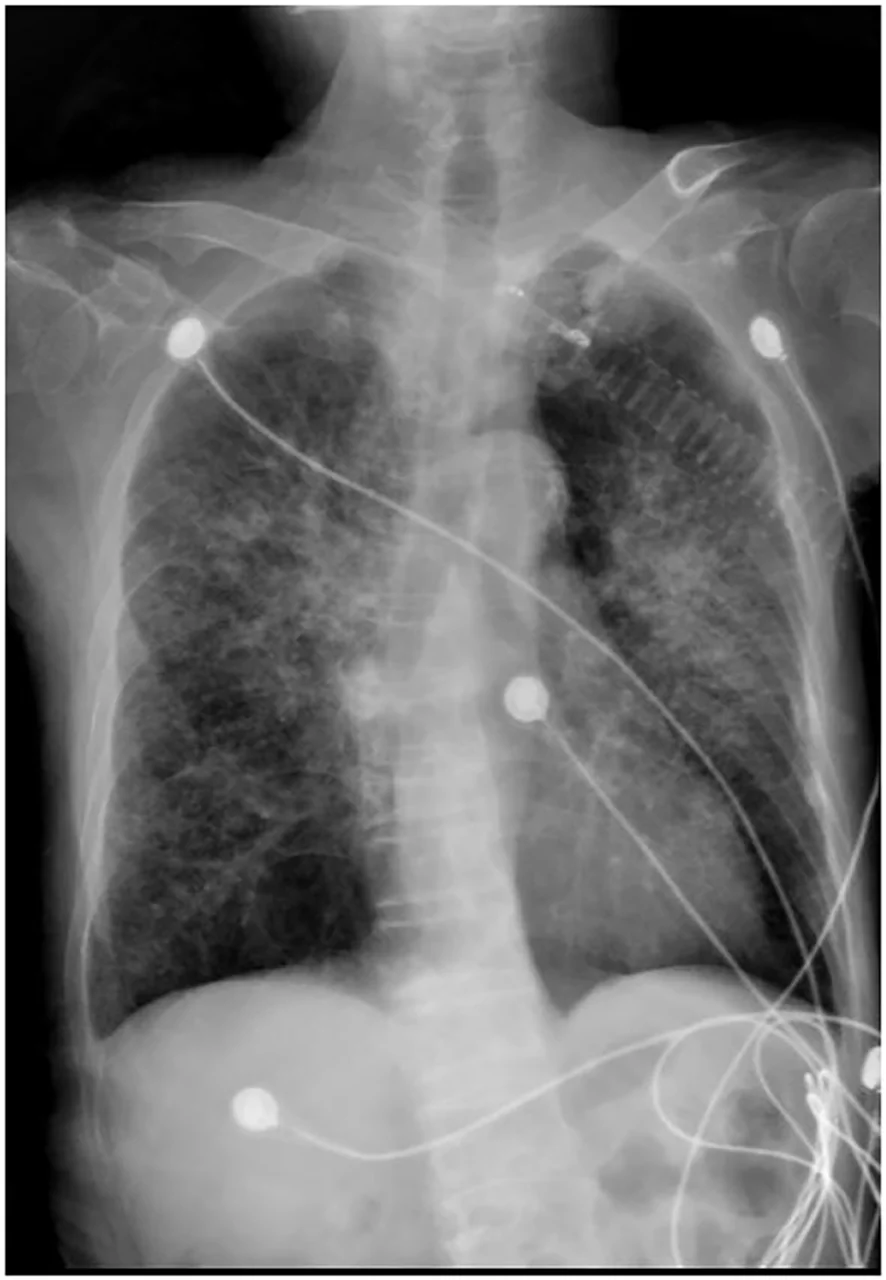
Chest radiograph showing bilateral diffuse pulmonary infiltration.

**Figure 2:**
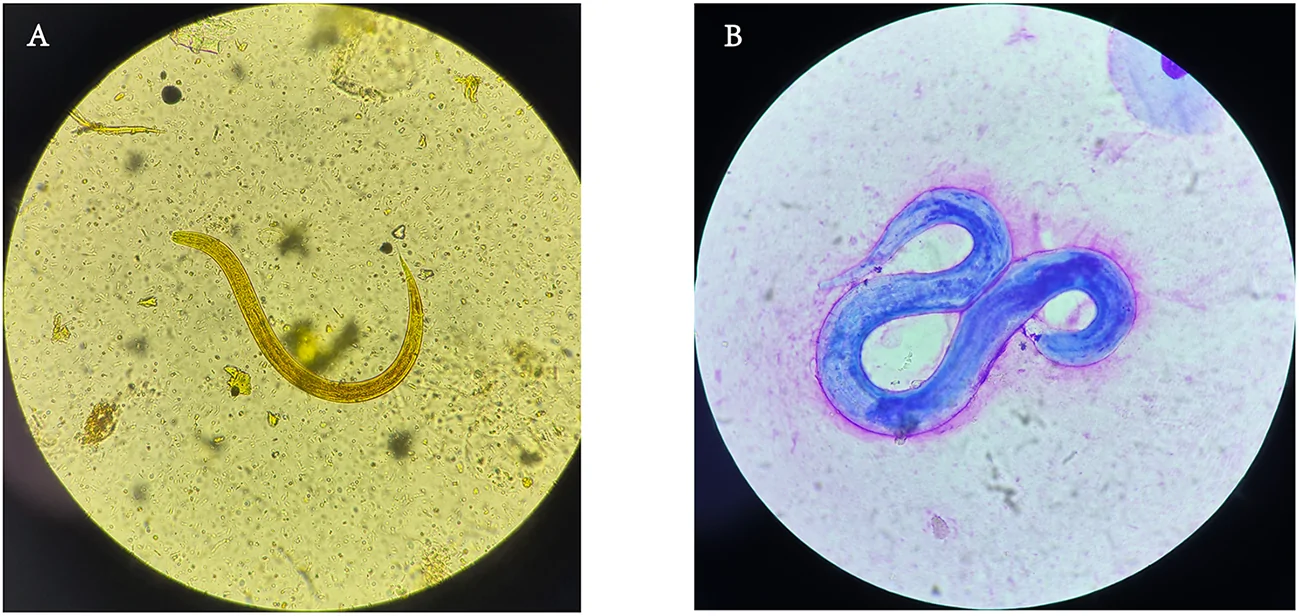
Strongyloides stercoralis detected in stool and sputum spicemens. **(A)** Rhabditiform larva of *S. stercoralis* in stool specimen (×400); **(B)** Filariform larva of *S. stercoralis* in Wright-Giemsa stained sputum (×1000).

Albendazole 400 mg/day was administered via nasogastric tube starting on January 29, and was switched to ivermectin 200 μg/kg/day on February 4.

Initially, based on the sputum mNGS results, the patient was treated with piperacillin/tazobactam, co-trimoxazole, voriconazole, and oseltamivir. Subsequently, due to inadequate infection control, the regimen was escalated to meropenem, linezolid, and caspofungin, and the antiviral agent was switched from oseltamivir to ganciclovir. The patient ultimately died on February 11, 2025, due to severe pneumonia, sepsis, and bone marrow suppression (bone marrow aspiration smear shown in **Figure 3**; the transfusion records summarized in **Supplementary Table 2**).

**Figure 3:**
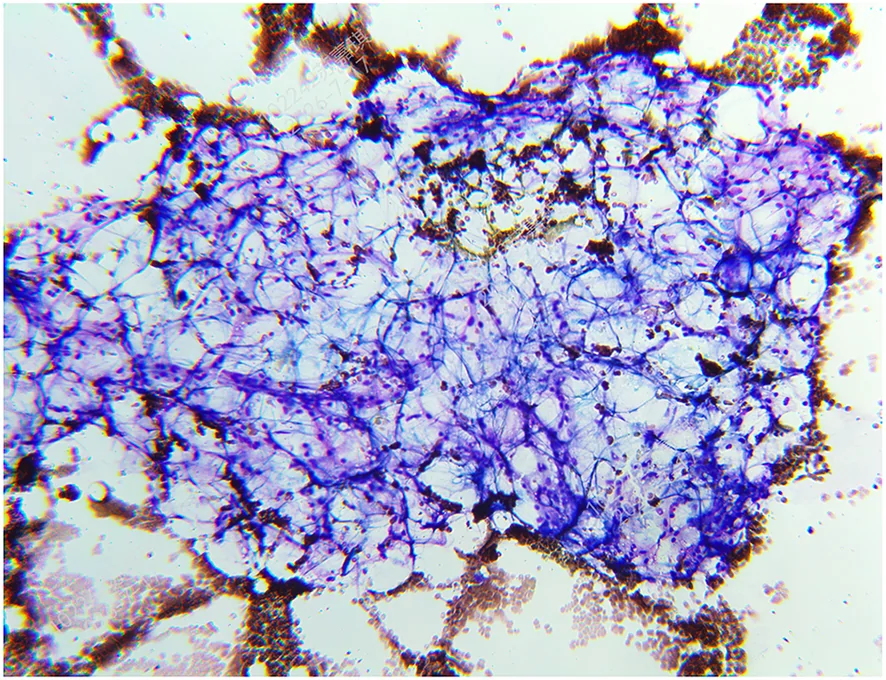
Bone marrow puncture smear examination on February 8th, 2025: Bone marrow particles showed an empty reticular framework wth markedly reduced hematopoietic cells (×100).

## Discussion

*S. stercoralis* is a soil-transmitted nematode widely distributed in tropical and subtropical regions, including humid areas of southern China (1). Chronic infection often presents as an asymptomatic chronic intestinal carrier state (2). However, when host immune function is compromised, the parasite can proliferate massively in the body through an accelerated autoinfection cycle, leading to SHS or even progressing to disseminated infection. SHS is a life-threatening condition with rapid progression, and mortality can reach up to 87% when there is dissemination (6). The present patient was an elderly male with SHS complicated by multiple underlying diseases, who ultimately died from severe pneumonia, sepsis, and bone marrow suppression due to progression of SHS. His diagnosis and treatment course provides an important clinical cautionary lesson.

In China, SHS cases have been occasionally reported, and immunosuppression is the most critical risk factor for the development of SHS (8–10). Glucocorticoids significantly promote the penetration of larvae through the intestinal wall into the bloodstream by suppressing Th2-type immune responses, reducing eosinophil recruitment, and impairing intestinal mucosal barrier function (11).

This patient had no obvious history of exposure to endemic areas or unhygienic living conditions, and the specific source of infection could not be traced. Although the patient had a history of COPD, diabetes mellitus, and cerebral infarction, he had not previously experienced severe gastrointestinal symptoms. Therefore, we speculate that the incomplete small bowel obstruction in this case may have been caused by diffuse intestinal wall edema induced by *S. stercoralis*. During treatment at the outside hospital, the patient received methylprednisolone therapy with glucocorticoid exposure. In addition, hyperglycemia resulting from diabetes and COPD are associated with systemic inflammation and immune dysfunction (7, 12). The combination of these multiple factors contributed to the development of SHS in this patient.

The diagnostic course of this case highlights the complexity of recognizing SHS in immunocompromised hosts. Unlike patients with diarrhea, this patient presented with intestinal obstruction as the initial symptom, resulting in prolonged absence of bowel movements, which reduced the probability of earlier detection of *S. stercoralis* in stool. On admission, the patient presented with bilateral diffuse pulmonary infiltrates, blood-streaked sputum, and irregular fever. Since mNGS results from January 27 and February 3 showed no parasitic sequences, the initial differential diagnosis focused on acute exacerbation of COPD, bacterial, fungal, and viral pneumonia. The diagnosis of SHS was ultimately established only after *S. stercoralis* larvae were detected in the stool smear on January 29 and upon retrospective re-examination of the sputum specimen from January 27. The limitations of mNGS for parasitic detection may stem from multiple factors: first, the nematode cuticle is rich in chitin, resulting in DNA extraction efficiency far lower than that for bacteria and fungi; second, human background DNA in respiratory specimens is extremely high, potentially masking low-load parasitic signals; third, larvae in BALF may represent only transiently expectorated organisms through the airway rather than alveolar colonization, leading to substantial sampling variability. This finding suggests that for immunocompromised hosts with unexplained pulmonary infiltrates combined with gastrointestinal symptoms, parasitic microscopic examination of stool and respiratory secretions should be actively pursued even when mNGS is negative, to avoid missed diagnosis from sole reliance on molecular testing.

Treatment of SHS requires simultaneous management of anti-parasitic therapy and co-infections. Ivermectin is currently recognized as the first-line agent, which enhances the permeability of glutamate-gated chloride channels in the parasite, leading to paralysis and death. Compared with albendazole, ivermectin achieves higher clearance rates for both adult worms and larvae, and has a longer half-life (13), facilitating repeated dosing in critically ill patients. Albendazole was initiated on January 29 due to the unavailability of ivermectin at our institution, and was switched to ivermectin on February 4 once ivermectin was obtained. However, SHS patients are at risk of secondary bacteremia due to intestinal bacterial translocation resulting from disruption of the intestinal mucosal barrier. Meanwhile, prolonged broad-spectrum antibiotics and immunocompromise increase the risk of invasive fungal infections. Both *Candida* and *Aspergillus* hyphae were observed in this patient’s sputum. Although blood cultures were negative, broad-spectrum anti-infective coverage for bacteria, fungi, and viruses was clinically warranted. This complex regimen of “anti-parasitic plus broad-spectrum anti-infective” therapy further aggravated drug toxicity and organ burden.

Despite aggressive anti-infective treatment and organ support, the patient ultimately died on February 11. Compared with the patient in our another case published on July 16, 2026 (10), this patient had more complex underlying diseases and more factors for poor prognosis, including advanced age, multiple comorbidities, malnutrition, delayed diagnosis, and disease progression to the terminal stage requiring endotracheal intubation and CRRT support. Literature reports indicate that once SHS progresses to the need for mechanical ventilation and renal replacement therapy, mortality increases significantly (14). Furthermore, bone marrow suppression developed during this patient’s hospitalization, which may have been related to severe infection itself, myelotoxicity from polypharmacy, or direct parasitic invasion, ultimately accelerating the progression to multiple organ failure.

## Clinical implications and limitations

The clinical implications of this case are as follows. First, for elderly patients with underlying diseases or glucocorticoid exposure who present with gastrointestinal symptoms, repeated and multi-sample parasitic screening is warranted. SHS should be suspected when pulmonary infiltrates coexist. Second, although mNGS offers significant advantages in detecting bacteria, fungi, and viruses, its sensitivity for parasites is limited. The conventional microscopy remains irreplaceable at present. Third, medical institutions should strengthen the stockpiling of anti-parasitic agents such as ivermectin to shorten the time to treatment initiation following SHS diagnosis. The limitations of this study include the inability to trace the specific dosage and duration of glucocorticoids administered at the outside hospital, which restricts precise assessment of the degree of immunosuppression. The lack of pathological confirmation of larval invasion into pulmonary parenchyma, making it difficult to fully differentiate SHS from disseminated strongyloidiasis. Future multicenter studies are needed to further validate the diagnostic value of mNGS in parasitic infections and to optimize treatment protocols for SHS.

## Conclusion

This case underscores that in immunocompromised patients with unexplained pulmonary infiltrates and gastrointestinal symptoms, active parasitic microscopic examination remains essential even when mNGS is negative. Early recognition and prompt anti-parasitic therapy, together with adequate drug availability, are critical to improving outcomes in SHS.

## Data Availability

The original contributions presented in the study are included in the article/**Supplementary Material**. Further inquiries can be directed to the corresponding author.
