# Supplementary material for: Case Report: mNGS-negative Strongyloides hyperinfection syndrome with small bowel obstruction as the initial manifestation in a glucocorticoid-exposed COPD patient: a fatal case
Source: Front Immunol. 2026 Oct 6;17:1940986. doi: 10.3389/fimmu.2026.1940986 (PMC13680226; doi:10.3389/fimmu.2026.1940986)
Supplement: Supplementary file 1 [file Table1.docx]

**Table S1. mNGS Results of Sputum (January 27, 2025) and BALF (February 3, 2025)**

| Sample | Pathogen Type | Species | Reads |
| --- | --- | --- | --- |
| Sputum | Virus | Human herpesvirus 1 (HSV-1) | 1,384,912 |
| Sputum | Virus | Cytomegalovirus (CMV) | 963,046 |
| Sputum | Virus | Human betaherpesvirus 6B (HHV-6B) | 30,522 |
| Sputum | Virus | Epstein-Barr virus (EBV) | 6,596 |
| Sputum | Bacteria | *Elizabethkingia meningoseptica* | 7,612 |
| Sputum | Bacteria | *Escherichia coli* | 4,847 |
| Sputum | Bacteria | *Enterococcus faecium* | 3,299 |
| Sputum | Bacteria | *Acinetobacter baumannii* | 2,201 |
| Sputum | Bacteria | *Moraxella catarrhalis* | 543 |
| Sputum | Fungi | *Pneumocystis jirovecii* | 91,101 |
| Sputum | Fungi | *Candida albicans* | 26,633 |
| Sputum | Fungi | *Aspergillus fumigatus* | 7,645 |
| BALF | Virus | Human herpesvirus 1 (HSV-1) | 648,080 |
| BALF | Virus | Cytomegalovirus (CMV) | 498,457 |
| BALF | Virus | Human betaherpesvirus 6B (HHV-6B) | 7,623 |
| BALF | Virus | Epstein-Barr virus (EBV) | 9,397 |
| BALF | Bacteria | *Enterococcus faecium* | 1,045,928 |
| BALF | Bacteria | *Acinetobacter baumannii* | 51 |
| BALF | Fungi | *Candida albicans* | 29,437 |

**Abbreviations:** BALF, bronchoalveolar lavage fluid; HSV-1, human herpesvirus 1; CMV, cytomegalovirus; HHV-6B, human betaherpesvirus 6B; EBV, Epstein-Barr virus. **Note:** No parasitic sequences were detected in either sample.
