# Supplementary material for: Case Report: mNGS-negative Strongyloides hyperinfection syndrome with small bowel obstruction as the initial manifestation in a glucocorticoid-exposed COPD patient: a fatal case
Source: Front Immunol. 2026 Oct 6;17:1940986. doi: 10.3389/fimmu.2026.1940986 (PMC13680226; doi:10.3389/fimmu.2026.1940986)
Supplement: Supplementary file 2 [file Table2.docx]

**Table S2. Red Blood Cell and Platelet Transfusions During Hospitalization**

| Date | Type | Transfusion Volume |
| --- | --- | --- |
| January 27th | LD-RBCs | 1 unit |
| January 30th | LD-RBCs | 1.5 units |
| February 3rd | LD-RBCs | 1 unit |
| February 5th | LD-RBCs | 1 unit |
| February 6th | LD-RBCs | 2 units |
| February 6th | LRA-Plt | 1 ATD |
| February 8th | LD-RBCs | 1.5 units |
| February 8th | LRA-Plt | 1 ATD |
| February 8th | LRA-Plt | 1 ATD |

**Abbreviations:** LD-RBCs: Leukocyte-Depleted Suspended Red Blood Cells; LRA-Plt: Leukocyte-Reduced Apheresis Platelet; ATD: adult therapeutic dose.
